# Outcome of Irrigation and Debridement after Failed Two-Stage Reimplantation for Periprosthetic Joint Infection

**DOI:** 10.1155/2018/2875018

**Published:** 2018-10-11

**Authors:** M. Faschingbauer, F. Boettner, R. Bieger, C. Weiner, H. Reichel, T. Kappe

**Affiliations:** ^1^Department for Orthopaedic Surgery, RKU, University of Ulm, Oberer Eselsberg 45, 89081 Ulm, Germany; ^2^Hospital for Special Surgery, 535 East 70th Street, New York, NY 10021, USA

## Abstract

**Introduction:**

Two-stage revision is the gold standard for the treatment of deep implant infection after knee or hip arthroplasty. Irrigation and debridement may be a treatment option for failed 2-stage revisions in cases where a reinfection occurs within 30 days or the symptoms exist not longer than 3 weeks and is appealing because of its low morbidity. We determined the incidence of recurrent infections following irrigation and debridement for failed two-stage revision hip and knee arthroplasty.

**Methods:**

We performed a single center retrospective review of periprosthetic hip and knee infections treated with a two-stage procedure from 2002 to 2010. All patients that subsequently underwent irrigation and debridement for a subsequent infection were selected for the current study.

**Results:**

440 two-stage revisions were performed between 2002 and 2010. Fifty-one two-stage revisions failed (11.6%). Nineteen failed two-stage revisions were treated with irrigation and debridement; 12 (63.2%) patients remained free of infection at follow-up (mean follow-up: 39 months; range, 24-90 months), infection persisted in 6 patients (31.6%), and 1 patient died (5.3%).

**Conclusions:**

Success rates of irrigation and debridement for failed two-stage procedures are similar to the success rates of irrigation and debridement in primary implant infections. According to the current paper, irrigation and debridement are an acceptable treatment for acute reinfections after failed two-stage revision if performed within the first 30 postoperative days after failed two-stage procedure or if symptoms are present for less than 3 weeks in the presence of a susceptible organism.

## 1. Introduction

Periprosthetic infection after total joint replacement is a devastating complication and occurs in up to 2% of primary joint replacements [1–5]. Treatment options include irrigation and debridement (I&D) with component retention [6–10], one-stage revision [11–13], or two-stage revision utilizing an antibiotic containing cement spacer [14–16].

Two-stage revision remains the golden standard treatment in the United States with infection-free survival rates of 80-100% [17–19]. An antibiotic containing cement spacer [14, 16] and intravenous antibiotics are routinely used for two-stage procedures. Zimmerli et al. [20] recommended two-stage revisions for patients with symptoms for more than 3 weeks or an index procedure performed more than 30 days ago.

Patients presenting with symptoms for less than 3 weeks or within 30 days of primary joint replacement without a sinus tract or radiographic evidence of component loosening can be considered for an I&D with retention of components in case of and a susceptible organism [21]. This approach is attractive because of its low-morbidity and cost effectiveness. However, failure rate of 7-79% has been reported and I&D is therefore in general considered less effective than two stage revision [6, 22].

There are much less clear recommendations for the treatment of recurrent infection following two stage revisions. Whiteside et al. [23] reported control of infection in 17 of 18 patients using aggressive I&D and intraarticular antibiotic infusion over 6 weeks after a failed two-stage treatment attempt.

The aim of the current study was to evaluate the clinical success rates of I&D after failed two-stage revision and to investigate the impact of the type of organism, patient age, general health condition (ASA), and comorbidities on treatment outcome.

## 2. Materials and Methods

The current study is a retrospective chart review of 440 two-stage revisions for periprosthetic knee or hip infection performed at one tertiary referral center between 2002 and 2010.

Patient demographics including age, gender, BMI, health status (ASA), the timespan between I&D, and the prior failed two-stage procedure, as well as number and type of prior surgeries, were recorded. The American Society of Anesthesiologists (ASA) physical status classification score was used as a proxy variable for health status. The types of organisms and sensitivities were documented for all procedures. Quinolone resistant Gram-negative bacteria, rifampicin-resistant Staphylococcus, Enterococcus, and Candida were classified as “difficult to treat” (DTT) in accordance of Winkler et al. [27].

The diagnosis of infection prior to the index two-stage procedure was based on clinical signs, blood work (ESR, CRP), positive synovial fluid aspiration, and intraoperative cultures (following the state of art [28]). The index two-stage revisions included removal of implants and bone cement (Stage I). All patients received a static antibiotic containing cement spacer (knees) or an articulating spacer (hip) as well as systemic antibiotics based on organism sensitivity for 2 weeks intravenously and for an additional 4 weeks orally. The articulating hip spacers were performed as mould-spacers with an endoskeleton (67%) or as handmade spacers (33%) [29]. The knee spacers were performed as static, handmade spacers with an endoskeleton [30]. Two weeks after stopping the systemic antibiotics successful eradication was confirmed by repeat joint aspiration. If the aspiration was negative, CRP remained less than 2 g/dL and there was no sinus tract and a new implant was inserted (Stage II).

Reinfection occurred in 51 patients (11.6%) of 440 two-stage revisions. If a reinfection occurred (diagnosis of reinfection followed the consensus criteria by Zmistowski et al. [1]) within 30 days after two-stage revision or patients presented with an acute reinfection (symptoms for less than 3 weeks), aggressive, if necessary repeated I&D was performed. Nineteen of 51 patients fulfilled the inclusion criteria (32 patients were treated with a second two-stage procedure). The mean age at I&D after failed two-stage procedure was 67.3 years (range, 45.2 – 84.5 years); there were 12 male and 7 female patients, 12 hips and 7 knees, 10 left and 9 right joints; the mean BMI was 29.6 kg/m^2^ (range, 21.5 to 36.4 kg/m^2^). Mean follow-up was 39 months (range, 24-90 months). At the time of each follow-up the absence of infection was evaluated by clinical criteria (no sinus tract, no swelling, no erythema, and no tenderness); if there was a reasonable suspicion of recurrent infection, an immediate work-up was done adhering to the guidelines by Parvizi et al. [28].

I&D was performed utilizing the preexisting incisions. After a thorough synovectomy and irrigation of the surgical site all removable components (hip: head and liner; knee: articular insert) were removed. Now the implant was cleaned and a through irrigation utilizing a minimum of 10 L of anti-infectious irrigation was performed. Finally the surgical field was covered with new drapes, new instruments were opened, and the surgeons and assistance were regloved and regowned. New mobile components were put in place and the wound was closed in layers in a usual way. If the soft tissues were not stable enough, 1-3 polyurethane sponges were inserted under the fascia or subcutaneously and were connected to a vacuum producing devise via tubes [31]; afterwards the wounds were still closed in layers under meticulous reconstruction and accurate adaptation of the tissue layers. A repeated I&D was performed 3-6 days later in a technique described by Kelm et al. [31]. The indication for a repetition of I&D was evidence of persistent microorganism intraoperatively, persisting drainage, no decrease of C-reactive protein within 6 days combined with clinical signs of persistent infection (overheat, reddening), or persisting sepsis.

All patients terminated the use of antibiotics two weeks after last surgery (“postoperative antibiotics”, Tables 2 and 4), and no suppression therapy was used.

Failure was defined as any additional surgery due to infection after hospital discharge. Only patients with a minimum follow-up of 24 months were included in the current study.

Descriptive statistics were calculated, including mean and frequency. Mann–Whitney test was used to determine differences between successes and failures. Chi-square tests were used to determine differences in proportions between dichotomous data.

All statistical analyses were performed using IBM SPSS Statistics software version 23 (Armonk, NY: IBM Corp.). A p-value of less than 0.05 was considered to be statistically significant.

## 3. Results

Of 19 patients who underwent I&D for failed two-stage revision, 12 (63.2%) patients were infection-free after a minimum follow-up of 24 months (mean 39 months, range 24-90 months). A recurrent deep infection occurred in 6 patients (31.6%) and one patient died (5.3%). 8/11 hips (72.7%) and 4/7 (57.1%) knees were infection-free after the minimum follow-up.

Patients with successful (Tables 1 and 2) and failed treatment (Tables 3 and 4) differed in regard to BMI (median, success group 31.5 kg/m^2^, reinfection group 25.5 kg/m^2^, p = 0.026), but there were no differences in age (p = 0.892), ASA grade (p = 0.989), and number of I&Ds (p = 0.243).

Causative organisms cultured at the time of I&D are reported in Tables 2 and 4. “Difficult to treat” organisms occurred in 6 cases. Two polymicrobial infections were observed. No statistically significant difference was found concerning the distribution of DTT-organisms or polymicrobial infections between the successfully treated and failed group.

## 4. Discussion

Periprosthetic infections are a devastating complication. Treatment options range from two-stage revision [22], one-stage exchange [11], or irrigation and debridement with retention of components [8]. There is an ongoing debate about the most appropriate treatment of an acute implant infection. While eradication rates are higher in two-stage procedures, quality of life and postoperative function might be better after one-stage and I&D procedures, respectively [32–34].

Eradication rates following revision of primary implant infections range from 61 to 100% for different treatment protocols [25, 32, 33]. However, literature on the treatment of recurrent infection is scarce. The current study reports the eradication rate utilizing I&D for recurrent periprosthetic infection within a time-window of 30 days or occurring symptoms less than 3 weeks in patients with failed two-stage revision for periprosthetic infection. In the present study similar eradication rates for an I&D (63.2%) after failed two-stage revision were shown compared to I&D in primary deep implant infection [6, 35, 36].

The current study has the following limitations. First, this is a retrospective study. Second, while the report is based on a large group of patients undergoing two-stage revision surgery cases, numbers in the current group of I&D for recurrent infection are too small to analyze the impact of the type of organism and its sensitivity on overall outcome of I&D. The numbers are also too small to make a differentiated analysis between hips and knees. Third, since patients did not undergo laboratory screening or recurrent aspiration, infections were assumed to be eradicated based on clinical criteria exclusively. Finally, the use of a polyurethane sponge with a vacuum-producing device during repeated I&Ds is not very well described in the literature. There are some reports using V.A.C-Instill with small patient-numbers, but only one study, in which also a deep sponge was placed and the wound was still closed anatomically [31] as in the current study. In our center the use of sponges with a vacuum system was left after 2010.

There are only a few papers that report on the treatment of failed treatment attempts of deep implant infections. There is no common sense of the best treatment after failed two-stage procedure. Stammers et al. [24] reported failure rates of 42% (8/19 knees) (Table 5) for repeat two stage revision after failed initial two stage treatment.

There is a study using a decision tree analysis to determine the best treatment (quality of life) after failed revision for deep implant infection. Wu et al. [37] expected the highest QoL utilizing arthrodesis following a failed two-stage revision in patients with total knee replacement. In a clinical review Sherrell et al. [25] reported failure rates of 34% in two-stage revisions for patients who underwent I&D followed by two-stage revision due to persisting infection. The authors assumed that the failure rate of 34% is higher than in patients who undergo two-stage revision only (Table 5).

Pagnano et al. [26] reported a reinfection rate of 18.7% (27/144 hips) after a first two-stage revision due to periprosthetic infection. The authors reported four treatment options after failed first two-stage revision: antibiotic suppression therapy, I&D, resection arthroplasty, or a second two-stage revision. Two of 3 patients did not need any further surgery after I&D and continuous oral suppressive antibiotic therapy. Sixteen patients were treated by resection arthroplasty after failed two-stage procedure. Three of these 16 patients (18.8%) had to undergo further surgeries for recurrence of infection. Eleven patients underwent a second two-stage revision, in 8 patients (72.7%), a recurrence of infection occurred and further surgeries were needed (Table 5).

In the current study, if necessary, repeat I&D was performed (range, 1-10). Kelm et al. [31] reported an eradication rate of 92.9% using the above mentioned protocol. However, the literature is inconsistent and some studies [38–40] report a higher failure rate with repeated I&Ds.

In the current study Staphylococcus was the predominant bacterium (52.6%; coagulase-negative staphylococci 42.1%, Staphylococcus aureus 10.5%). This finding is in line with current literature [41, 42]. “Difficult to treat”-organisms (DTT) occurred in 6 patients. Winkler et al. [27] described rifampicin-resistant Staphylococci (Patient 1), quinolone-resistant gram-negative bacteria (Patient 18), Enterococci (Patient 5, 9, 12 and perished patient), and Candida as DTT-organisms. No differences concerning the success of I&D were observed in the present study according to the presence or absence of DTT-organisms. One polymicrobial infection (Patient 5 and 12) occurred in each group. It is not clarified yet if the classification of DTT-infections should include polymicrobial infections. Only 3 patients showed the same organism at the time of two-stage revision and I&D (2x MRSE: Patient 1 and 2; 1x Staph. aureus: Patient 7). Two patients (Patient 14 and 15) developed methicillin-resistance (Staph. Epidermidis). Zmistowski et al. [43] differentiate between recurrence of infection (same organism at the failed two-stage procedure and at the renewed flare-up infection) and “new” reinfection with a change of microorganism. They showed a persistent infection with the same organism in 31.5%. Haddad et al. [42] also reported a change of organism in reinfection in 3 of 4 (75%) patients and specified these patients as reinfected compared to the fourth patient with a “persisting infection”. Kraay et al. [44] actually described a 100% “new” infection-rate after failed two-stage procedure (28 patients). Triantafyllopoulos et al. [45] also showed more “new” infections than persistent infections with the same microorganism. In the current study 84.2% of patients showed “new” infection. Due to the majority of “new” infections it can be concluded, as Zmistowski et al. [43] and Triantafyllopoulos et al. [45] do, that the host status with all possible comorbidities may be a major factor. The control and improvement of comorbidities cannot be overstated as the mentioned studies describe a high vulnerability of the host for a “new” infection with a high Charlson Comorbidity Index. For patients with a high Charlson Comorbidity Index as a vulnerable host and an increased risk of perioperative complications the concept of I&D (defined as an acute reinfection within above mentioned time window) is a feasible option of treatment. On the other hand Zmistowski et al. [43] showed the only independent predictor of persistent periprosthetic joint infection was a primary infection with Staphylococcus in general, and MRSA in particular. These numbers can be shown in the current study as well as all three persistent infections occurred in patients with a Staphylococcus-infection. At the current study all persistent infections (n = 3; same microorganism within the failed two-stage procedure and the reinfection) are in the “infection free” group after I&D. It is unclear why the success rate in persistent infections was 100%. Three reasons of this circumstance could be as follows: first, the basis of the mature biofilm (prosthesis) was removed during the two-stage procedure and it might be that the revival of a mature biofilm at the new prosthesis was sufficiently stopped by a timely I&D. Second, the numbers of patients are too small. Third, the follow-up period is too short. The antibiotic therapy used in the current study follows the guidelines by Osmon et al. [20].

In summary, the failure rate of I&D for acute recurrence of infection (within 30 days of symptoms or 3 weeks of two stage procedure) following failed two-stage treatment was 31.6% in the present study. Therefore the concept of I&D after failed two-stage procedure might be an option in acute reinfections more than in persistent infections. The success rate is comparable to I&D for infection of a primary joint replacement. Careful indication (see criteria), meticulous surgical debridement, and close cooperation between the microbiologist, infectious disease doctor, and surgeon are recommended.

## Figures and Tables

**Table 1:** Successful cases and demographics.

**Patient**	**Age (years)**	**Gender**	**Joint**	**Side**	**ASA grade**	**Follow up (months)**	**BMI**	**Comorbidities**
1	73.5	male	Hip	R	3	33	34.2	HTN, A

2	46.5	male	Hip	R	3	34	32.1	HTN, SM, A

3	68.3	male	Hip	L	3	28	30.9	HTN, CKF

7	74.4	female	Hip	L	3	63	36.4	HTN, post enterovesical fistula

8	63.2	male	Hip	L	2	77	21.5	SM, DM

9	64.3	female	Hip	R	2	24	35.4	HTN, CHF

11	52.9	female	Hip	L	2	90	34.3	-

12	75.9	female	Knee	R	3	24	32.8	HTN, absolute arrhythmia in atrial fibrillation

13	45.2	male	Knee	R	2	25	29.3	Post ORIF femur

14	76.2	male	Knee	R	3	30	29.8	HTN, CHD, CHF, CKF, absolute arrhythmia in atrial fibrillation

18	84.5	male	Hip	L	4	28	24.2	HTN, CHD, CKD, post apoplexy, morbus parkinson, chronic cystitis

19	67.4	female	Knee	R	3	27	29.7	Bipolar psychosis, lumbar spinal syndrome

DM = diabetes mellitus, HTN = arterial hypertension, CHD = coronary heart disease, CHF = chronic heart failure, CKF = chronic kidney failure, COPD = chronic obstructive pulmonary disease, SM = smoker, A = alcohol (> 20 g/d), DA = drug abuse

**Table 2:** Successful cases with organism type, resistogram, and antibiotics; failed two-stage revision was done at a tertiary referral center; prior surgeries (column 2) were done in external clinics.

Patient	Revision surgeries prior to failed two-stage revision	Organism type: failed two-stage revision	Antibiotics preoperative	Number of surgeries (I&D)	Organism type: I&D	Antibiotics postoperativ	Resistant against
1	Two-stage revision	MRSE	Va, Ti, Ri	1	MRSE	Va, Ti, Ri	Ox, Ri, Ci, Ge, Cl, Im

2	-	MRSE	Le, Ri	7	MRSE	Le, Va, Ri	Ox, Ci, Ge, Cl

3	Revision due to bursitis intertrochanterica	Staph. capitis	Le, Ri	1	Strepto. intermedius	Le, Ri	Cl

7	-	Staph. aureus	-	8	Staph. aureus	Le, Fl, Ri	-

8	Bursectomy, Exchange of cup		Le, Ri	7	MRSE	Va, Cl, Ri	Ox, Ce, Im

9	Soft tissue revision	Strepto. agalactiae	Fl, Ri	1	Enterococcus faecalis	Fl, Am, Ri	Ge, Cl, Ce

11	-		Le, Ri	2	Staph. aureus	Le, Ri	-

12	Two-stage revision		Le, Ri	5	(1) Enterococcus faecalis (2) Peptostrepto. magnus	Le, Amp, Ri	(1) Ge, Cl (2) -

13	ORIF (tibiaplateau fracture)	MRSE	-	6	Escherichia coli	Le, Ce, Ri	-

14	-	Staph. epi	Le, Ri	11	MRSE	Va, Ri	Ox, Ge, Cl, Ce, Va, Im

18	Two-stage revision	Staph. aureus	-	7	Escherichia coli	Me, Ri	Le, Ci,

19	3x two-stage revision	Strept. simulans	Le, Ri	5	Escherichia coli	Le, Ri	-

MRSE = methicillin-resistant staphylococcus epidermidis, Ox = oxacillin, Ri = rifampicin, Le = levofloxacin, Ci = ciprofloxacin, To = tobramycin, Fl = flucloxacillin, Ge = gentamicin, Cl = clindamycin, Ce = cefuroxime, Va, = vancomycin, Li = linezolid, Im = imipenem, Ti = tigecycline, Am = amoxicillin, Amp = ampicillin, Mo = moxifloxacin, Me = meropenem

**Table 3:** Failed cases and demographics.

**Patient**	**Age (years)**	**Gender**	**Joint**	**Side**	**ASA grade**	**Follow up (months)**	**BMI**	**Comorbidities**
5	76.6	female	Hip	R	3	55	23.5	Post cerebellar infarction, osteoporosis, post humerus-fracture

6	70.8	male	Hip	L	3	29	27.8	DM, HTN, CHD, CHF, CKF, post quadruple coronary artery bypass, both sided carotid artery stenosis

10	62.4	male	Hip	R	2	29	26.3	SM, A, post transient ischemic attack

15	61.9	male	Knee	L	2	49	29.8	HTN, post phlebothrombosis

16	78.5	female	Knee	L	3	35	23.5	-

17	62.1	male	Knee	L	4	24	24.7	DM, HTN, CHD (bio heart valve, pacemaker), CKD (IgA nephropathy), A, post phlebothrombosis

DM = diabetes mellitus, HTN = arterial hypertension, CHD = coronary heart disease, CHF = chronic heart failure, CKF = chronic kidney failure, COPD = chronic obstructive pulmonary disease, SM = smoker, A = alcohol (> 20 g/d), DA = drug abuse

**Table 4:** Failed cases.

Patient	Revision surgeries prior to failed two-stage revision	Organism type: failed two-stage revision	Antibiotics preoperative	Number of surgeries (I&D)	Organism type: I&D	Antibiotics postoperative	Resistant against
5	Inlay exchange (multiple dislocations), multiple surgeries due to infection	(1) Staph. aureus	Le, Ri	5	(1) MRSE	Va, Ri	(1) Ox, Ci, Cl
		(2) Staph. haemolyticus			(2) Enterococcus faecium		
							(2) Ci, Ge, Cl

6	Exchange of cup	MRSE	Cl, Ri	5	Enterobacter cloacae	Le, Ri	-

10	Exchange of cup (3x)	Staph. epidermidis	-	2	-	Le, Ri	-

15	Arthroscopy with synovectomie	Staph. epidermidis	Le, Ri	1	MRSE	Va	Ox, Ge, Cl, Ce, Im

16	-	Candida albicans	-	3	MRSE	Cl	Ox, Ge, Ce, Im

17	2x two-stage revision, 1x tibial exchange	Enterobacter cloacae	Me	7	MRSE	Va, Mo, Ri	Ox, Ge, Ce, Im

MRSE = methicillin-resistant staphylococcus epidermidis, Ox = oxacillin, Ri = rifampicin, Le = levofloxacin, Ci = ciprofloxacin, To = tobramycin, Fl = flucloxacillin, Ge = gentamicin, Cl = clindamycin, Ce = cefuroxime, Va, = vancomycin, Li = linezolid, Im = imipenem, Ti = tigecycline, Am = amoxicillin, Amp = ampicillin, Mo = moxifloxacin, Me = meropenem

**Table 5:** Failure rates after failed revisions due to periprosthetic infection.

**Author**	**First Revision**	**Second Revision**	**Failure-rate**
Stammers et al. [24]	Two-stage revision	Two-stage revision	42% (8/19)

Sherrell et al. [25]	I&D	Two-stage revision	34% (28/83)

Pagnano et al. [26]	Two-stage revision	Resection arthroplasty	19% (3/16)
	Two-stage revision	Two-stage revision	73% (8/11)

Current study	Two-stage revision	I&D	32% (6/19)

## Data Availability

The data used to support the findings of this study are available from the corresponding author upon request. The paper was accepted by SICOT as a poster presentation at the 37th Orthopaedic World Congress in Rome, 2016
